# Incidence of community-acquired infections of lower airways among infants

**DOI:** 10.1016/j.rppede.2015.10.005

**Published:** 2016

**Authors:** Ana Luisa Oenning Martins, Deisy da Silva Fernandes Nascimento, Ione Jayce Ceola Schneider, Fabiana Schuelter-Trevisol

**Affiliations:** a Universidade do Sul de Santa Catarina (Unisul), Tubarão, SC, Brazil; b Universidade Federal de Santa Catarina (UFSC), Florianópolis, SC, Brazil; c Centro de Pesquisas Clínicas do Hospita Nossa Senhora da Conceição, Tubarão, SC, Brazil

**Keywords:** Pneumonia, Bronchiolitis, Child, Epidemiology, Risk factors

## Abstract

**Objective:**

To estimate the incidence of community-acquired infections of the lower respiratory tract and the risk factors associated with its occurrence in infants, in their first year of life.

**Methods:**

A prospective cohort study of infants who were followed up during the first 12 months of life. Interviews were conducted with their mothers, and children were clinically monitored bimonthly to investigate the occurrence of the incidence density of community-acquired infections of the lower respiratory tract. Cox regression analysis was used to estimate the crude and adjusted relative risk of the variables associated with the outcome.

**Results:**

The mean age of the mothers was 26 years, 62% of them had more than 11 years of schooling, and 23.5 were at risk of social exclusion regarding economic income. The incidence density of pneumonia and bronchiolitis were, respectively, 0.51 and 3.10 episodes per 100 children-months. Children who had low birth weight (<2500g) were 5.96 (95%CI 1.75-20.40) times more likely to have pneumonia than infants weighing 2500g or over.

**Conclusions:**

The incidence of acute lower respiratory tract infection in children was similar to that found in other studies. Only low birth weight was an independent risk factor for the occurrence of pneumonia.

## Resumo

**Objetivo:**

Estimar a incidência de infecções comunitárias de vias aéreas inferiores e os fatores de risco associados à sua ocorrência em lactentes em seu primeiro ano de vida.

**Métodos:**

Estudo prospectivo de coorte com lactentes que foram acompanhados durante os primeiros 12 meses de vida. Foram feitas entrevistas com as mães e as crianças foram clinicamente monitoradas bimestralmente para investigar a ocorrência da densidade de incidência de infecções comunitárias das vias aéreas inferiores. A análise de regressão de Cox foi usada para estimar o risco relativo bruto e ajustado das variáveis associadas com o desfecho.

**Resultados:**

A média de idade das mães foi de 26 anos, 62% tinham mais de 11 anos de escolaridade e 23,5 estavam em risco de exclusão social em relação à renda. A densidade de incidência de pneumonia e bronquiolite foi, respectivamente, 0,51 e 3,10 episódios por 100 crianças-mês. Crianças com baixo peso ao nascer (<2.500 gramas) foram 5,96 vezes (IC95% 1,75-20,40) mais propensas a ter pneumonia do que crianças com peso de 2.500 gramas ou mais.

**Conclusões:**

A incidência da infecção aguda das vias aéreas inferiores em crianças foi semelhante à encontrada em outros estudos. Apenas baixo peso ao nascer foi um fator de risco independente para a ocorrência de pneumonia.

## Introduction

Acute respiratory tract infections (ARIs) are a leading cause of morbidity and mortality among children. In 2010, infectious diseases caused 58% of deaths globally among children younger than 5 years. Pneumonia, diarrhea and malaria accounted for one-third of deaths in this age group.^1^ In Latin America, respiratory infections were responsible for over 80,000 deaths of children per year, 40% of which occurred in Brazil.^2^ The World Health Organization (WHO) considers that bronchiolitis and pneumonia are the most important epidemiological components of ARIs in early childhood.^3^

Bronchiolitis is an acute infection of the small airways that primarily affects young infants, often those aged between 2 and 24 months. The disease follows a seasonal pattern, with peaks during the winter in temperate climates, and during the rainy season in tropical climates.^4^ Pneumonia is a leading cause of morbidity and mortality among children younger than five years, with 95% of cases occurring in developing countries.^3^ Previous studies have listed the risk factors for acquiring respiratory infections, among which are socioeconomic factors (low household income, poor parental education, and high number of persons per household), early cessation of breastfeeding, low birth weight, malnutrition, passive smoking, and daycare attendance.^5^^,^ 6

In this context, the aim of this study was to estimate the incidence of community-acquired lower respiratory tract infections, and the risk factors associated with its occurrence in infants up to one year old in Tubarão, southern Brazil.

## Method

This study was approved by the Research Ethics Committee of the University of Southern Santa Catarina (code number 12.035.4.01 III) on April 27, 2012.

This is a prospective cohort study. The municipality of Tubarão is home to 96,284 residents. According to the Information Technology Department of the National Health System (DATASUS), around 2000 children are born in Tubarão yearly (average for the last 10 years), above 80% per year, on average, in the maternity of *Hospital Nossa Senhora da Conceição* (HNSC). The HNSC is reference center and it is the only hospital that provides neonatal intensive care in the region. It is a “Baby-friendly hospital” since 2001.

It is estimated that one-third of infants have at least one episode of lower respiratory tract infection in their first year of life.^7^ The sample size calculation considered the following: exclusive breastfeeding is the main protective factor for reducing lower respiratory tract infections (LRTIs); reduction of LRTI provided by exclusive breastfeeding in about 65%^8^; power of 80%; alpha error of 5%; and 95% significance level. A 20% addition was made to cover possible losses to follow-up, totaling a minimum sample size of 106 subjects.

Data were collected between June 2012 and September 2013 from mothers of newborn babies at the *Hospital Nossa Senhora da Conceição*. After getting a written consent form, the participants were interviewed to collect data regarding prenatal care, delivery details, and demographic and socioeconomic characteristics of the family. Mothers who agreed to participate were given a health diary to make weekly notes about their child's clinical data. The health diaries were collected at each medical appointment, and new ones were handed out. Childcare was provided to all children included in the study, with bimonthly scheduled medical appointments for a one-year period. During these visits, clinical follow-up data were collected, and confirmation of data provided by mothers in the health diaries was made. Six medical appointments were provided to each child over a one-year period. The children were seen by physicians who were pediatricians and professors at the medical school at two outpatient clinics run by the University of Southern Santa Catarina.

In this study, pneumonia and bronchiolitis were diagnosed by a physician when the baby's mother sought health care. Symptoms associated with pneumonia were cough, fever, and radiographic changes to confirm the diagnosis, whereas symptoms associated with bronchiolitis were tachypnea, cough and wheezing with presence or absence of fever and coryza. The data were collected during the pediatric visits and reported by the mothers.

**Figure 1:**
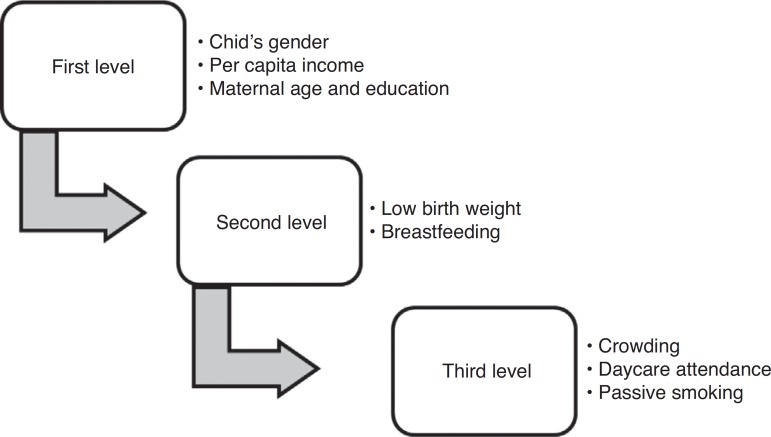
Description of hierarchical analysis for acute respiratory tract infections.

The Open Source Epidemiologic Statistics for Public Health (OpenEpi), version 2.3.1 was used to calculate the sample size. The collected data were entered into EpiData program version 3.1 (EpiData Association, Odense, Denmark), and statistical analysis was performed using the Statistical Product and Service Solutions software (SPSS) for Windows, version 20 (IBM SPSS Statistics, Chicago, IL, USA). The outcome incidence was calculated as incidence density rate, expressed as the number of events per person-time. Cox regression analysis was used to calculate the crude and adjusted relative risk for outcome variables. The significance level was set at 5%. Multivariate analysis was used for adjustment of confounding factors, according to the hierarchical model proposed by Victora et al.,^9^ as shown in Fig. 1.

## Results

**Figure 2:**
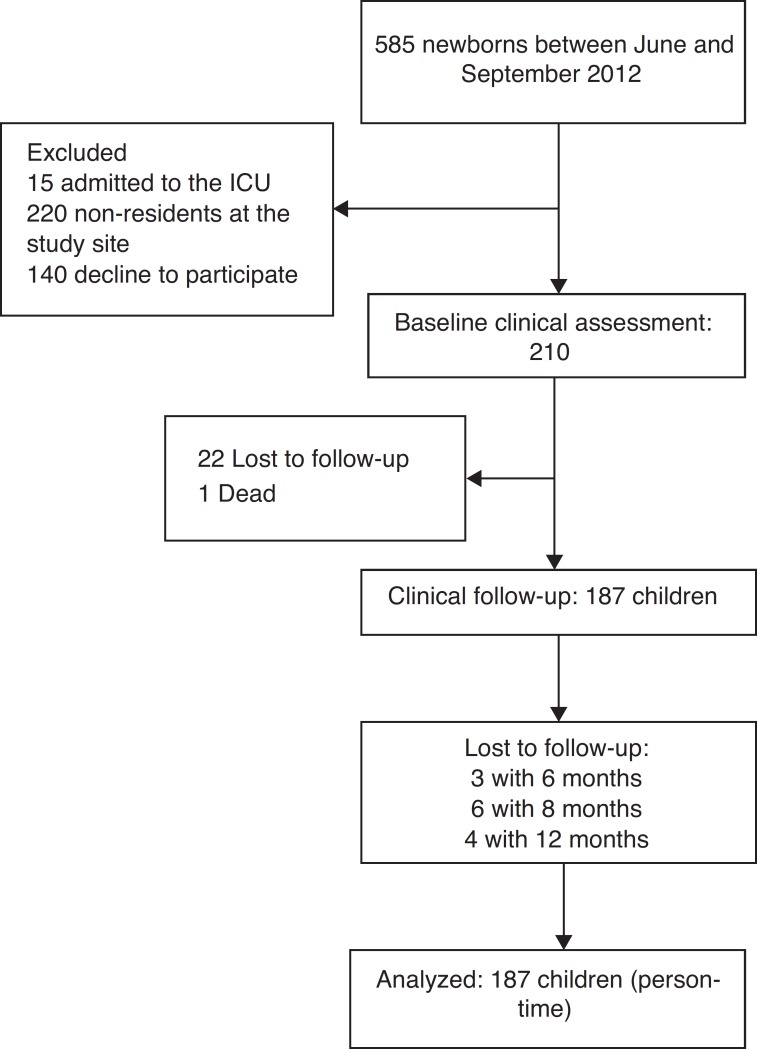
Flowchart of study participants.

Between June 2012 and September 2013, 210 interviews were conducted with nursing mothers. Fig. 2 shows a flowchart demonstrating the selection of study participants. The sample with complete data consisted of 187 infants (89.0%), of whom 87 (46.5%) were male. The mean maternal age was 26 years (range, 14-45 years). Regarding the socioeconomic status, the median household income was BRL 1866.00, whereas calculating 60% of the median per capita income, which is a parameter used by the Brazilian Institute of Geography and Statistics (IBGE) to indicate relative poverty, resulted in BRL 248.80. With regard to education, 62.0% (95%CI: 54.5-69.0) of the nursing mothers had more than 11 years of school attendance, and 65.8% (95%CI: 59.1-72.6) of the respondents lived with more than three people in the same home. Only 27 (14.4%) infants were exclusively breastfed until six months of age.

During the study period, there were 11 cases of pneumonia (6.5%, corresponding to 0.51 episodes per 100 children-month) and 71 cases of bronchiolitis (42.3%, corresponding to 3.10 episodes per 100 children-month). Only 1 infant had pneumonia on two occasions, at eight and ten months of age. Regarding bronchiolitis, 14 children had one recurrent episode, 4 children had two recurrent episodes, and 1 child had four episodes of bronchiolitis during the first year of life. Of all children who were diagnosed with ARI, only 1 was hospitalized for pneumonia in the first month of life. Table 1 presents the data regarding the exposure to ARIs and perinatal risk factors (Fig. 1).

**Table 1:** Perinatal risk factors and exposure to acute respiratory tract infections (*n*=187).

	*n*	%	95%CI^a^
Birth weight <2500g	17	9.1	5.4-13.4
Gender Male	87	46.5	39.6-53.5
Passive smoking	53	28.3	21.9-34.8
Daycare attendance	28	15.0	10.2-20.9
Exclusive breastfeeding <6 months	89	47.6	40.6-54.5

a CI, confidence interval.

Using Cox regression analysis, low birth weight was associated with the occurrence of pneumonia. Children who were born weighing less than 2500g were 5.83 times more likely to develop pneumonia than normal birth weight infants. No significant association was found between pneumonia and breastfeeding, daycare attendance, passive smoking, and other studied variables. No significant associations between bronchiolitis and the study variables were found. Table 2 shows the relative risk of the variable adjustments using a hierarchical model.

**Table 2:** Relative risk of variables regarding pneumonia and bronchiolitis adjusted according to the hierarchical model for estimating incidence density.

Risk factors	Adjusted RR (95%CI)	*p*-value	Adjusted RR (95%CI)	*p*-value
	Pneumonia		Bronchiolitis	
*First level*				
Gender		0.454		0.332
Male	1.58 (0.49-5.22)		0.78 (0.47-1.29)	
Per capita income^a^		0.452		0.606
<BRL 248.80	0.44 (0.05-3.68)		1.16 (0.65-2.07)	
School attendance		0.139		0.640
0-11 years	0.20 (0.02-1.67)		0.88 (0.51-1.51)	
Maternal age		0.913		0.907
<20 years	1.01 (0.90-1.12)		1.00 (0.96-1.04)	

*Second level*				
Birth weight		0.006		0.592
<2500g	5.96 (1.75-20.4)		0.78 (0.31-1.94)	
Exclusive breastfeeding		0.234		0.859
<6 months	0.45 (0.12-1.68)		1.06 (0.54-2.07)	

*Third level*				
Persons per household		0.212		0.448
>3	3.73 (0.47-29.4)		0.83 (0.51-1.34)	
Daycare attendance		0.643		0.143
Yes	1.38 (0.35-5.49)		1.52 (0.87-2.65)	
Passive smoking		0.960		0.779
Yes	0.93 (0.56-1.54)		1.08 (0.65-1.78)	

a Cut-off point: 60% of median income.

None of the maternal risk factors (household income, age, education level, and number of persons per household) were associated with ARIs in the present study.

After relative risk adjustments according to the hierarchical model, low birth weight was associated with the occurrence of pneumonia. Children who were born weighing less than 2500g were 5.96 (95%CI 1.75-20.4) times more likely to develop pneumonia compared with those weighing 2500g or more.

## Discussion

In the present study, there was a high cumulative incidence of bronchiolitis (42.3%) and pneumonia (6.5%), which highlights the relevance of these respiratory morbidities among children. These findings are consistent with several other studies that consider ARIs as common events during infancy and childhood. However, published studies show variation in the incidence of pneumonia and bronchiolitis, probably due to different criteria for defining the incidence of these occurrences. In a study conducted on 936 children, Aldous et al.^10^ reported that 32% of the study participants had at least one episode of ARI in the first year of life. Flaherman et al.^11^ carried out a retrospective cohort study on 123,264 children in California and reported that 16.7% of children developed bronchiolitis before the second year of life. A recent study conducted in South Africa found that 87.4% of children under 5 years of age had at least one episode of bronchiolitis or pneumonia in an 18-month period. Of these children, 10.5% had two ARI episodes, and 1.7% had three episodes.^12^ Bates et al.^13^ reported that 49.5% of infants who participated in their study in Nepal had bronchiolitis or pneumonia.

The incidence density of bronchiolitis (3.1 episodes per 100 children-months) and pneumonia (0.51 episodes per 100 children-months) in the present study can be compared with findings from different authors. In a recent systematic review, Rudan et al.^14^ found a mean incidence density of pneumonia (1.83 episodes per 100 children-month) among children under five years of age in low and middle income countries. In another study conducted on children under five years of age, an incidence density of pneumonia of 2.4 episodes per 100 children-month was found in developing countries.^3^ Weber et al.^15^ reported that the incidence density of bronchiolitis in Gambia was 7.3 episodes per 100 children-month among children aged 19-25 months. In a cohort study conducted on children from birth to three years old, Broor et al.^16^ reported that the incidence density of ARIs was 4.5 episodes per 100 children-month in infants under one year of age, without discriminating between bronchiolitis and pneumonia.

In the current study, low birth weight was an independent risk factor for the occurrence of pneumonia. Children who were born weighing less than 2500g were 5.96 times more likely to get pneumonia than those weighing 2500g or more. Prietsch et al.^2^ found that the prevalence of ARI was 10% higher in children with low birth weight compared with normal birth weight infants. Low birth weight has been associated with severe pneumonia and increased risk of mortality in several studies.^17^^,^ 18 Using a case-control study, Nascimento et al.^19^ found that low birth weight increased twice the risk of hospitalization for pneumonia. The same author explains that children with low birth weight have decreased immune response and impaired pulmonary function due to the smaller diameter of the large airways and easier obstruction of peripheral airway than normal birth weight infants.^20^ However, the causal relationship between low birth weight and the occurrence of pneumonia is complex, representing a cumulative effect of a number of nutritional and non-nutritional prenatal exposure.^21^

Several studies have shown that breastfeeding reduces the risk of morbidity and hospitalizations for ARI.^22^^,^ 23 In a meta-analysis conducted on up to 2-year-old children in developed countries, Bachrach et al.^7^ found that breastfeeding was a protective factor: exclusive breastfeeding for more than four months can reduce by 70% the risk of hospitalization for respiratory diseases. In the current study, no association between breastfeeding and the occurrence of ARIs was observed. In studies conducted by Nascimento et al.^19^ and by Pavić et al.,^24^ breastfeeding also showed no protective effect against these infections. A possible explanation for these conflicting results is that breastfeeding reduces the disease severity and risk of hospitalization for ARIs, but it does not eliminate the occurrence of these infections.

Only 15% of children who participated in this study attended daycare centers, and they had no greater risk of ARIs than those who did not attend, according to the analyses performed. In a case-control study, Macedo et al.^22^ found no significant association between daycare attendance and increased hospitalization for ARI. These results are in disagreement with most of the studies that associate daycare attendance with a significant increase in the incidence and prevalence of ARIs.^25^^,^ 26 Possibly, they attended daycare centers with adequate ventilation, hygiene, and care conditions, which provided similar conditions to those at home. Moreover, the results may have been influenced by the low percentage of children attending daycare centers. In addition, the length of daycare attendance was not investigated, which could also explain the lack of association between these variables.

Regarding passive smoking, 28.3% of children were exposed to secondhand smoke for living with smokers in this study. However, this variable was not a significant risk factor for ARI, which contradicts most studies that link smoking with the occurrence of bronchiolitis.^27^^-^ 29 Welliver et al.,^30^ however, found no association between passive smoking and acute bronchiolitis, but with subsequent recurrent wheezing. With regard to pneumonia, many studies have also found no association between passive smoking and the occurrence of the disease.^19^^,^ 23 ^,^ 26 In addition, this study has neither quantified the children's tobacco intake nor measured the frequency of exposure to tobacco, which may explain the lack of association. None of the maternal risk factors (household income, age, education level, and number of persons per household) were associated with ARIs in the present study. The high percentage of mothers with more than 11 years of school attendance (62%) may have favored the absence of association between poor education and occurrence of outcomes in this cohort.

This study has some limitations that should be kept in mind when interpreting the results. The follow-up period was rather short to examine whether the results had statistical significance. The lack of environmental quality assessment of the air was another limitation of the study. It is well known that air pollution is an important risk factor for the development of ARIs, but children who participated in this study lived in cities where there was no systematic monitoring of air quality and exposure assessment. It should also be mentioned that there is another private maternity hospital in the municipality for those who can afford to pay for it or have a health care plan. Thus, the sample included in this study may have presented a homogeneous socioeconomic status, which could have influenced the absence of association between family income and the occurrence of outcomes.

The incidence density of pneumonia and bronchiolitis were 0.51 and 3.10 episodes per 100 children-months, respectively. There was no significant correlation between the occurrence of bronchiolitis and the variables tested.

Based on the results from this study, it can be concluded that children who were born weighing less than 2500g have higher risk of pneumonia than normal birth weight infants, which highlights the need for further studies to identify factors related to low birth weight and preventive mechanisms to prevent the occurrence of the disease.
